# Enhanced Safety and Efficiency of Ambulatory Cardiology Admissions: A Quality Improvement Initiative

**DOI:** 10.1097/pq9.0000000000000726

**Published:** 2024-05-09

**Authors:** Mary C. McLellan, Mariam Irshad, Katherine C. Penny, Michelle Rufo, Sarah Atwood, Heather Dacey, Christina M. Ireland, Sarah de Ferranti, Theresa Saia, Anna C. Fisk, Susan F. Saleeb

**Affiliations:** From * Acute Cardiac Care Unit, Benderson Family Heart Center, Boston Children’s Hospital, Boston, Mass.; † Boston Children’s Hospital, Boston, Mass.; ‡ Cardiology Clinic, Benderson Family Heart Center, Boston Children’s Hospital, Boston, Mass.; § Department of Cardiology, Benderson Family Heart Center, Boston Children’s Hospital, Boston, Mass.; ¶ Ambulatory Cardiology Division, Benderson Family Heart Center, Boston Children’s Hospital, Boston, Mass.; ‖ Cardiology Nursing and Patient Care Operations, Benderson Family Heart Center, Boston Children’s Hospital, Boston, Mass.; ** Cardiac Intensive Care Unit, Benderson Family Heart Center, Boston Children’s Hospital, Boston, Mass.

## Abstract

**Background:**

Pediatric cardiac patients have experienced evolving illnesses progressing to instability while awaiting inpatient admission from ambulatory settings. Admission delays and communication breakdowns increase the risk for tenuous patients. This quality improvement initiative aimed to improve safety and efficiency for patients admitted from an ambulatory Clinic to the Acute Cardiac Care Unit (ACCU) using standardized communication and admission processes within one year.

**Methods:**

An admission process map, in-clinic nurse monitoring, and communication pathways were developed and implemented. A standardized team handoff occurred via virtual huddle using illness severity, patient summary, action list, situational awareness, and synthesis. Escalation of care events and timeliness were compared pre- and postimplementation.

**Results:**

There was a reduction of transfers to the intensive care unit within 24 hours of ACCU admission from 9.2% to 3.8% (*P* = 0.26), intensive care unit evaluations (without transfer) from 5.6% to 0% (*P* = 0.06), and arrests from 3.7% to 0% (*P* = 0.16). After the pilot, clinic nurses monitored 100% of at-risk patients. Overall mean time from admission decision to virtual huddle decreased from 81 to 61 minutes and mean time to admission from 144 to 115 minutes, with 41% (n = 33) arriving ≤ 60 minutes (goal). The COVID-19 pandemic negatively affected admission timeliness while safety metrics remained optimized.

**Conclusions:**

Implementing a standardized admission process between the Clinic and ACCU enhanced safety by reducing admission wait time and escalation of care post-admission. Sustainable, reliable handoff processes, in-clinic monitoring, and standardized admission processes were established. The pandemic hindered admission efficiency without compromising safety.

## INTRODUCTION

Children and adults with congenital, arrhythmic and acquired heart disease can encounter unplanned admissions from the ambulatory cardiology clinic (Clinic) to the Acute Cardiac Care Unit (ACCU). While most patients are stable when awaiting admission, fragile patients with evolving cardiac instability have experienced rare but severe decompensation events before or shortly after admission.

*Sentinel event case*: An adolescent with underlying muscular dystrophy and no prior cardiac dysfunction had numerous recent presentations to the emergency room with abdominal complaints and fatigue. An echocardiogram in the clinic noted new, severe left ventricular dysfunction with an ejection fraction of 15%, prompting the decision to admit. The heart rate in the clinic at check-in was 124 bpm. Following physician-to-physician sign-out, the patient awaited admission for ~4 hours in the Clinic waiting room without further vital signs obtained. Immediately upon ACCU arrival, the patient’s heart rate was 204 bpm with a complex ectopic atrial tachycardia leading to a syncopal event. The patient decompensated further and required urgent transfer to CICU for initiation of extracorporeal membrane oxygenation (ECMO). Communication silos and lack of additional in-clinic assessment resulted in unseen clinical change, ineffective triage priority, and delayed treatment.

**Fig. 1.**
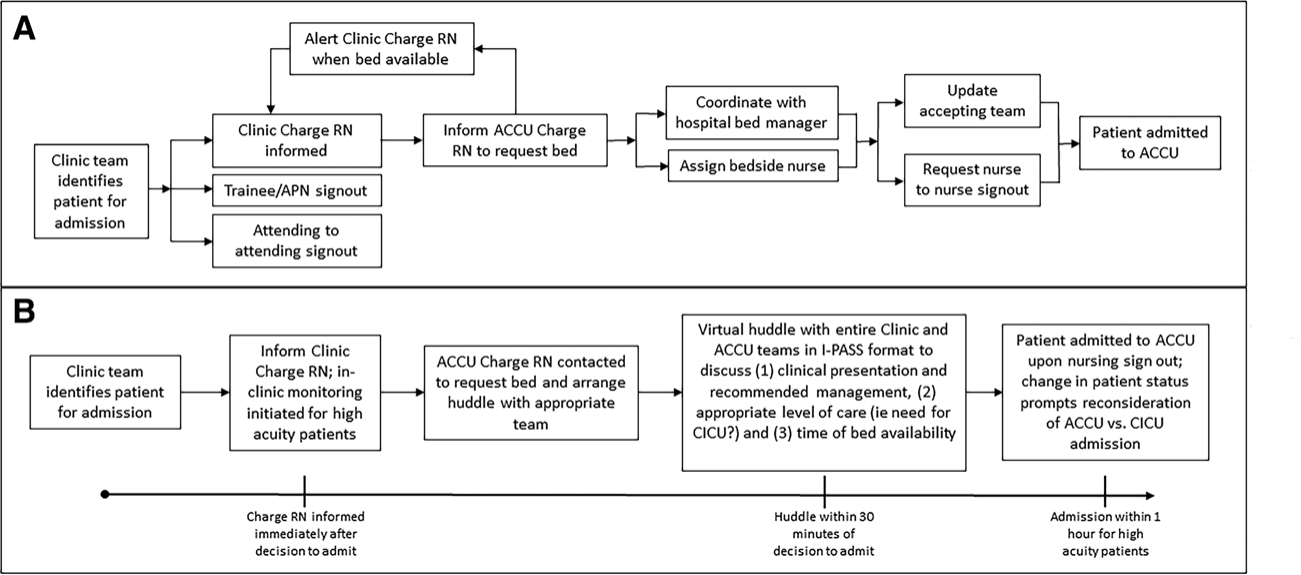
Admission process pre- and post-implementation of quality improvement work. Before QI efforts, the admission process (A) relied on multiple parallel communications. The streamlined admission process after (B) QI efforts relies on prompt, closed-loop communication across teams within targeted timeframes, establishment of in-clinic nurse monitoring when warranted, and ongoing triage of admission location. APP, advanced practice provider; I-PASS, illness severity, patient summary, action list, situational awareness, and synthesis; RN, registered nurse.

Delays in the admission process and breakdowns in communication increase the risk for vulnerable patients. Handoff communication around admissions from the Clinic occurred through several parallel conversations between physicians, nurses, hospital bed managers, advanced practice clinicians, and trainees (Fig. 1A). In-clinic vital sign monitoring was not routinely practiced preadmission, leaving changes in patients’ clinical conditions unrecognized. Any of these described gaps could result in a quick, unanticipated patient transfer to the Cardiac Intensive Care Unit (CICU) shortly after ACCU admission.

Standardized patient handoff communication and processes would decrease care failures, improve the clarity of transition responsibilities, and minimize distractions and interruptions during handoff.^1^ A mnemonic, I-PASS, was developed from best handoff practices as a framework for verbal and electronic handoff between residents caring for inpatients and admissions from the emergency room.^2–4^ I-PASS represents the following handoff elements: I-illness severity, P-patient summary, A-action list, S-situational awareness and contingency planning, and S-synthesis by the receiver.^3^ The landmark study by Starmer et al in 2014 assessed I-PASS among residents across 9 institutions and reported a relative reduction in rates of medical errors by 23%, preventable adverse events by 30%, and near misses and nonharmful errors by 21%, while not increasing handoff duration. A variety of hospital care areas and disciplines have adopted I-PASS for handoff.^5–7^ Handoffs occurring from the ambulatory to inpatient units have been insufficiently studied, forming the basis of this quality improvement (QI) work.

The interdisciplinary QI workgroup aimed to improve safety and efficiency for inpatient admissions from the Clinic through standardized closed-loop communication handoff processes, monitoring and surveillance of patients’ preadmission, and reducing wait times before ACCU admission. Specific aims, to be achieved within one year, included:

- Reduction of escalation of care events within 24 hours of ACCU admission from 9% to 0%.
- Introduction of VH communication between the Clinic and ACCU teams; aim to complete the VH within 30 minutes from the decision to admit high-risk (later termed “acuity”) patients.
- Reduction in wait time for high-risk patient admissions from 81 to ≤ 60 minutes from decision to admit.
- Introduction of in-clinic nurse monitoring for all high-risk patients before ACCU admission.

## METHODS

### Setting

This effort occurred within a large Heart Center at a freestanding, urban, academic, pediatric, quaternary, 477-bed hospital. The Ambulatory Pediatric Cardiology Clinic in this hospital is high-volume and fast-paced, with >25,000 annual visits and approximately 60–70 unplanned admissions from the Clinic to the ACCU annually. Many unplanned admissions stem from urgent (within 24–48 hours) clinic add-on appointments, a common practice instead of referral to the emergency room.

In September 2016, the Heart Center initiated a QI project to implement team-based standardized handoff using I-PASS within and across the ACCU, CICU, and Cardiovascular Operating Room. The Institute for Health Improvement Model for Improvement and the institution’s High-Reliability Organization principles provided the project’s framework.^8,9^ The project aligns with the hospital’s Clinical Handoff Guidelines Policy, which defines clinical handoff as an interactive communication process to provide accurate patient care information using I-PASS.^10^ The Heart Center I-PASS QI project team identified clinicians across the Heart Center to form an interdisciplinary workgroup to improve the patient safety of Clinic admissions. The workgroup included nursing and physician leadership from the Clinic, ACCU, CICU, cardiac catheterization procedural unit, Cardiac Anesthesia, and Emergency Department.

**Fig. 2.**
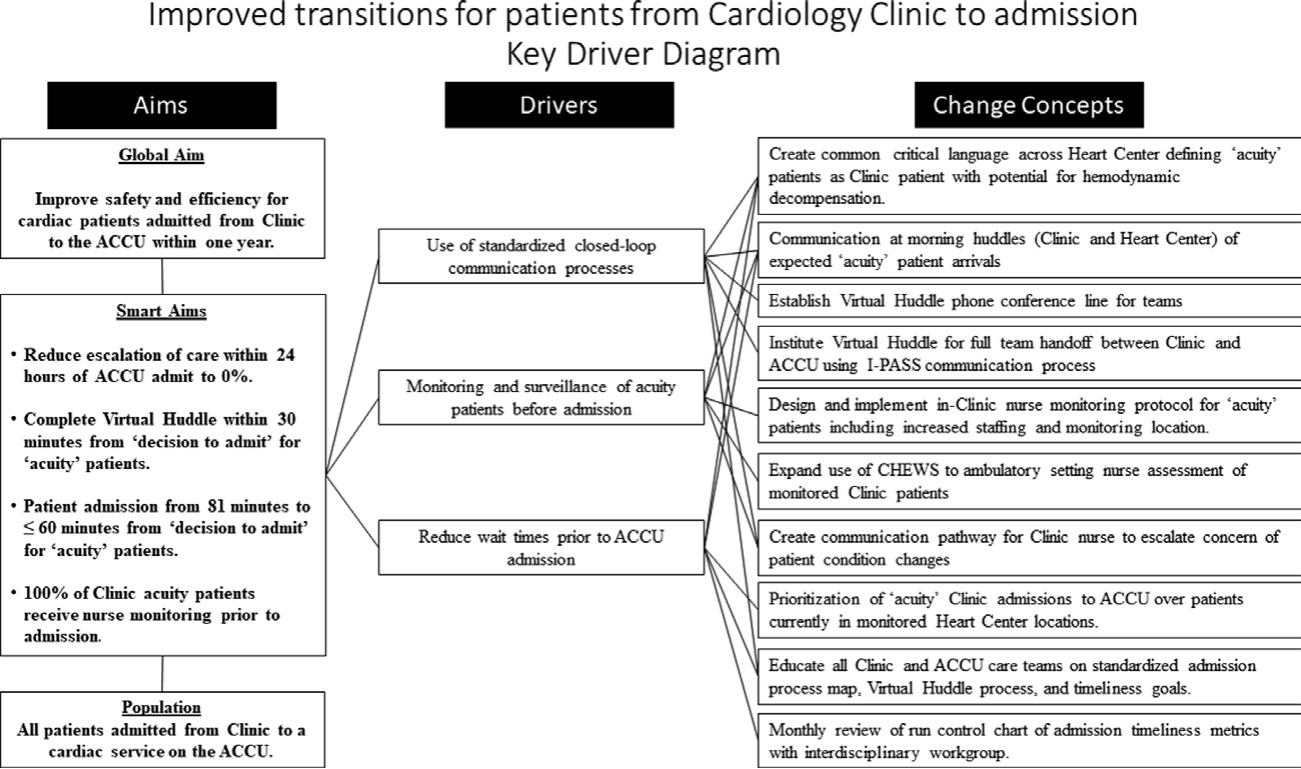
Key Driver Diagram for patients from Cardiology Clinic to admission.

The workgroup reviewed a historical cohort (May 2018 to May 2019) of unplanned admissions from the Clinic to understand and describe the frequency and severity of escalation of care events [intensive care unit (ICU) evaluation, ICU transfer, sepsis huddle, cardiac and/or respiratory arrest] in Clinic and within the first 24 hours of ACCU admission from Clinic. Historical data from Clinic charge nurse tracking (March 2017 through December 2017) described baseline wait times for patient admissions (average 144 minutes) as a reference to establish goal timeliness targets. The workgroup brainstormed to decrease wait times and increase patient safety peri- and post-admission. Solutions were translated into the project’s charter, aim, objectives, and QI metrics. Adoption of the Heart Center I-PASS QI Project’s key driver diagram occurred after modification applicable to this care environment (Fig. 2).

### Interventions

The term “acuity patient” was developed as a critical language to define a sick ambulatory patient requiring priority admission over other patients awaiting admission in monitored settings (ie, post-catheterization recovery). Potential acuity patients, usually added to the clinic schedule the previous day, are discussed during the morning Clinic huddle and the Heart Center capacity huddle for situational awareness and bed planning.

Following the decision to admit, Clinic and ACCU charge nurses communicate with each other, and their respective teams plan team handoff. Structured I-PASS handoff communication between Clinic and admitting teams occurs via a “Virtual Huddle” (VH) (Fig. 1B), using a single, streamlined telephone or video conference line with a designated number and passcode. The phone number and passcode appear on admission process maps in all Heart Center clinical areas and are programmed into hospital-based portable telephones. Using an I-PASS structured handoff checklist during VH ensures a complete description of patient illness and team agreement of appropriate admission location.

The clinic created space and designated nursing resources for nurse monitoring of preadmission acuity patients with vital signs performed at least every 30 minutes. The Clinic adopted the Children’s Hospital Early Warning Score (CHEWS; **See figure, Supplemental Digital Content 1,** which shows the CHEWS reference tool. http://links.lww.com/PQ9/A546), a deterioration assessment tool for pediatric inpatient areas, for monitoring.^11,12^ Nurses utilize patients’ vital signs and assessments to calculate a CHEWS score and reference a corresponding escalation of care algorithm. In the algorithm, a score of 0–2 directs standard of care, 3–4 (elevated) directs increased assessment frequency and team discussion for intervention plans, and ≥5 (critical) adds bedside evaluation by provider, attending physician notification, and consideration of Rapid Response Team activation and/or CICU transfer.^11,12^ Acuity patients presenting desaturated or with arrhythmias receive continuous oxygen saturation and heart rate monitoring with audible alarms for surveillance. The Clinic team sets vital sign parameters and findings requiring escalation defined for the nurse to prompt reevaluation by the Clinic team and re-decision on ACCU versus CICU level care.

### Study of Interventions

Process metrics for patients admitted from the Clinic to the ACCU included the percentage of acuity patients that received in-clinic nurse monitoring, timeliness of admission and VH from admit decision, and completion of I-PASS team handoff. A hospital-wide I-PASS initiative included handoff observation of I-PASS adherence with at least 10 monthly handoff observations or 50% of all handoffs (whichever was less). I-PASS champions entered observations into a secure database and ran control charts to provide clinical areas with monthly trends.^10^ Workgroup members reported I-PASS adherence results at workgroup meetings. However, data were not specific for Clinic to ACCU handoffs or included for project data. Outcome metrics for patient safety were measured by the absence of escalation of care events (previously defined) peri-admission and within 24 hours postadmission. Heart Center systems monitored the balancing metrics, including impact on throughput, increased clinician time for handoff, added clinician resources, and reported back at workgroup meetings.

**Fig. 3.**
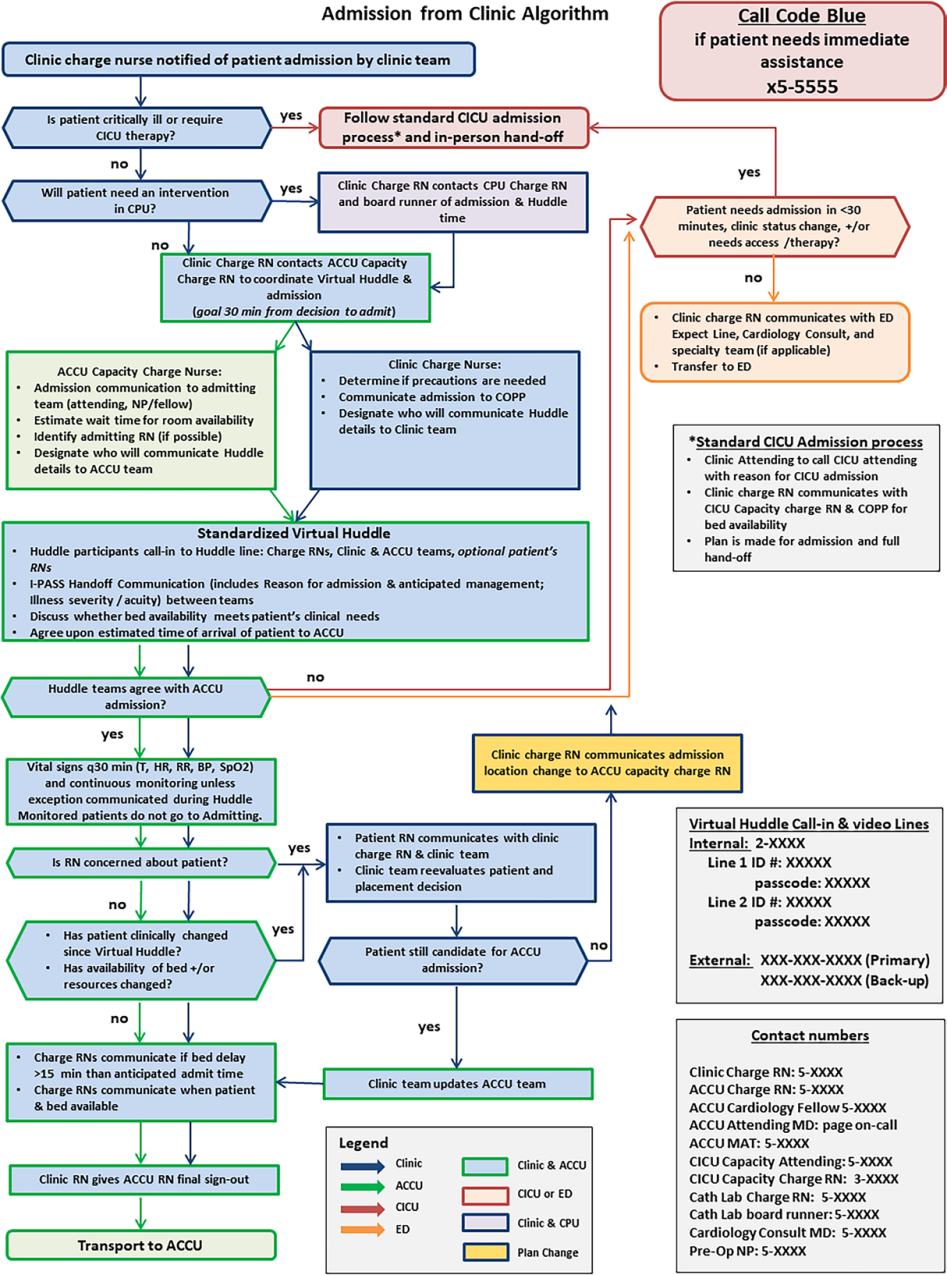
Process map for admission from the Clinic to the ACCU, CICU, Cardiac Procedure Unit, or Emergency Department. I-PASS, Illness severity, patient summary, action list, situational awareness, and synthesis; RN, registered nurse.

Between January 2019 and May 2019, the workgroup utilized four modified Delphi rounds to develop a standardized process map for admissions from the Clinic (Fig. 3). From May to June 2019, the case report forms and data collection processes were developed and implemented. A 2-week pilot period was conducted in June 2019 and debriefed, further refining the VH process. Data collection processes were modified, and additions were made to the case report form for data clarity using 3 Delphi rounds over 2 months.

### Measures

Data entered into a secure, institutionally-maintained REDCap database from June 2019 to August 2021 included in-clinic nurse monitoring adherence, timeliness of VH and admission, reasons for delayed admissions, CHEWS scores in Clinic and upon ACCU admission, escalation of care within 24 hours of ACCU admission, and unexpected in-hospital death. Patient demographic data include gender, age, primary cardiac diagnosis, and admission reason. The historic cohort served as a pre-implementation comparison group. The workgroup provided an educational series, an I-PASS checklist, and reference materials on the new handoff and admission processes. Occurrences of handoff-related errors were monitored in the hospital’s safety event reporting system. The full project implementation was in June 2019.

### Analysis

Categorical variables were counted and summarized as percentages, mean values were calculated for continuous variables and process control charts were analyzed quarterly in workgroup meetings for timeliness trends. Fisher exact tests were calculated to compare pre and postcohorts.

### Ethical Considerations

The scope of this study was for operational and QI and, therefore, the study met institutional review board exemption criteria.

## RESULTS

Table 1 describes demographics, primary cardiac disease, and admitting locations for pre- and postimplementation patient groups. During the intervention period, the Clinic sent 93 patients for admission. Median age at admission was significantly younger during implementation (2.6 versus 10.6 years, *P* = 0.002). Most (95%, n = 88) of patients were admitted to Cardiology Medicine services, and the remainder were admitted to other medical services.

**Table 1.** Clinical Characteristics and Admitting Location of Pre- and Postintervention Cohorts.

Characteristic	Historic Cohort, n (%)	Implementation, n (%)	*P* value
No. admissions	59	93	
Sex, male	44 (76)	52 (56)	0.02
Median age at admission, y (range)	10.6 (0–65.3)	2.6 (0–51)	0.002
**Primary Cardiac Diagnosis** Single ventricle Cardiomyopathy/ventricular dysfunction Left-to-right shunt, congestive failure Conotruncal lesion Primary valve disease Left-sided obstructions Pulmonary vein stenosis Kawasaki disease Arrhythmia Pulmonary hypertension Pericardial effusion	20 (34) 3 (5) 6 (10) 10 (17) 6 (10) 2 (3) 0 1 (2) 6 (10) 4 (7) 1 (2)	24 (26) 17 (18) 13 (14) 12 (13) 11 (12) 8 (9) 3 (3) 3 (3) 1 (1) 1 (1) 0	0.01
**Admitting Location** ACCU CICU Emergency department noncardiac inpatient unit	54 (91) 4 (7) 1 (2)	80 (86) 8 (9) 3 (3) 2 (2)	0.69

Compared with pre-implementation, ACCU admissions (n = 80) transferred to the ICU within 24 hours of admission decreased from 9.2% to 3.8% (*P* = 0.26), CICU evaluations (without transfer) from 5.6% to 0% (*P* = 0.06), arrests from 3.7% to 0% (*P* = 0.16), and sepsis huddles from 1.8% to 1.2% (*P* = 1). Pre-implementation, there were two infant arrests shortly after arrival to the ACCU (22 and 11 minutes, respectively), with one patient who did not survive to discharge. The patient that survived had a hypercyanotic spell (unrepaired tetralogy of Fallot) and received respiratory resuscitation before CICU transfer. The patient who did not survive (Stage 1 palliation of complex single ventricle physiology) experienced a bradycardiac arrest leading to extracorporeal CPR and was withdrawn from support 2 days postarrest. After implementation, no patients admitted from the Clinic experienced an arrest. Pre-implementation, all the ACCU’s Clinic admissions that transferred to the CICU (n = 5) needed urgent care interventions. In comparison, two of the three CICU transfers postimplementation were electively sent to the CICU for increased surveillance.

After the first quarter of implementation, 100% of acuity patients received in-clinic nurse monitoring and documented CHEWS scores over six consecutive quarters. The mean last in-clinic CHEWS score was 1.7 (range 0–6, n = 64), and the first ACCU CHEWS was 1.3 (range 0–3, n = 88). The mean difference between the CHEWS scores of patients (n = 64) was 0.34 (95% CI [0.004–0.783], *P* = 0.27).

**Fig. 4.**
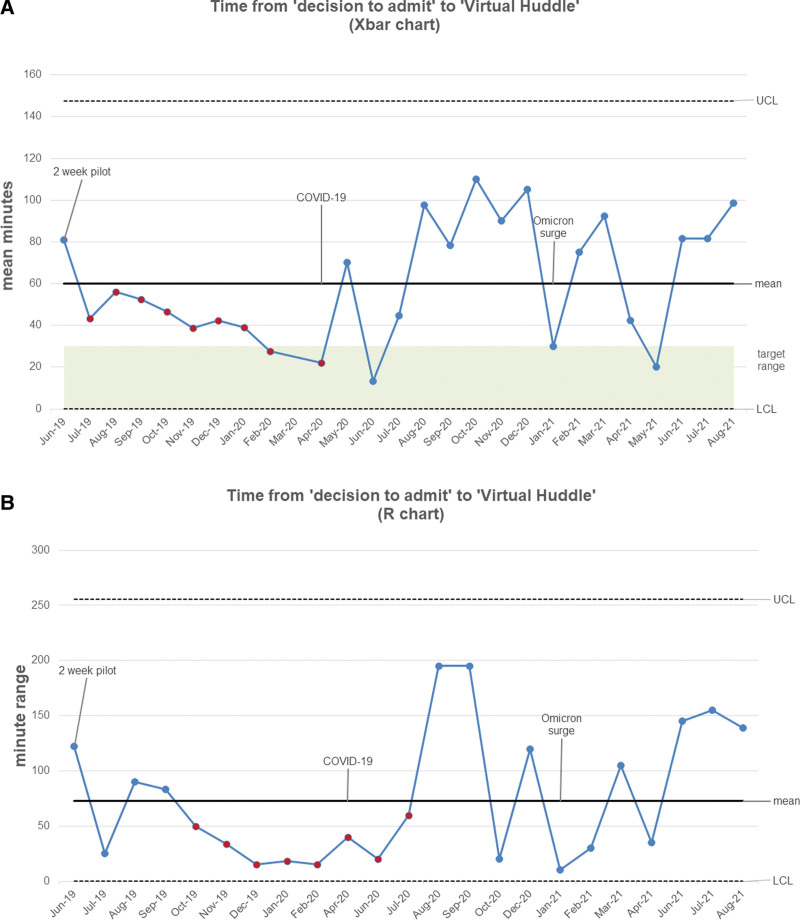
Timeliness of decision to admit to virtual huddle. X bar (a) R Control Chart (b) of timeliness from the decision to admit Clinic patient to team VH handoff.

During the 2-week pilot in June 2019, the mean time from admission decision to VH was 81 minutes and 60 minutes for the entire intervention period (Fig. 4). From June 2019 to March 2020, the mean time from admission decision to VH decreased to 48 minutes with a sustained shift through April 2020 (target goal 30 minutes). There was an increase in time to VH starting during the COVID-19 pandemic (average of 70 minutes) and an omicron variant surge (average of 65 minutes).

**Fig. 5.**
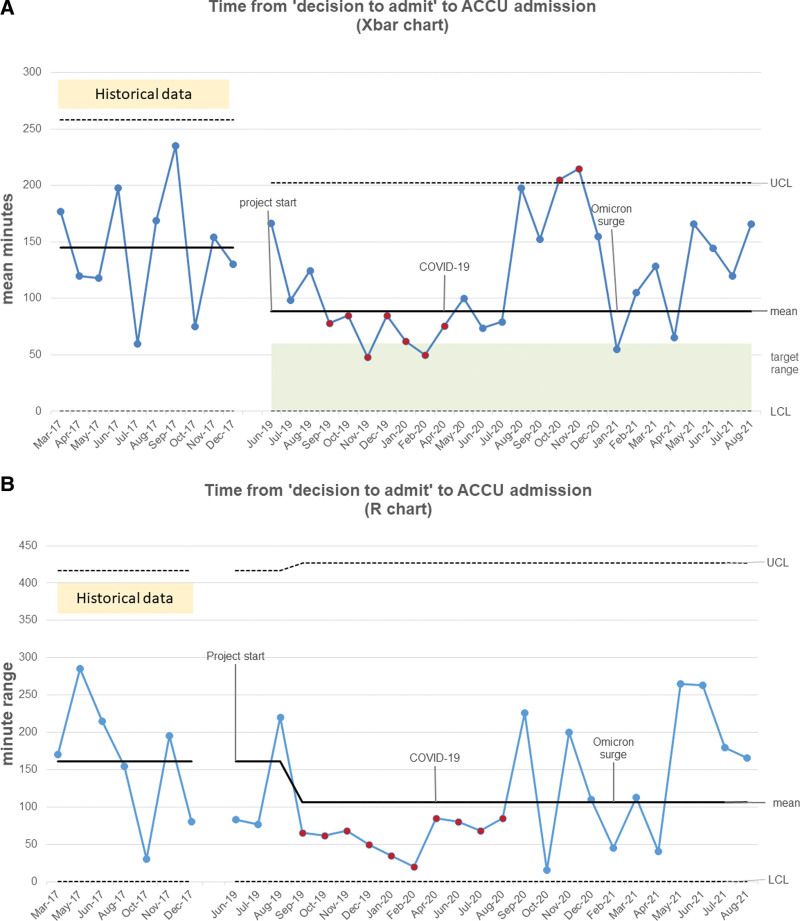
Timeliness of admission to Acute Cardiac Care Unit. X bar (a) R Control Chart (b) of timeliness from the decision to admit Clinic patient to ACCU Admission.

Historical data (March–December 2017) established the average time to admission at 144 minutes (Fig. 5). After implementation, the average time to admission steadily decreased to 80 minutes with a sustained shift through July 2020 (target goal 60 minutes). Admission timeliness and coordination were negatively impacted during the start of the COVID-19 pandemic (spring 2020) due to pandemic protocols (ie, single patient per room). Subsequent COVID-19 surges, such as with the Omicron variant during spring 2021, repeatedly affected timeliness metrics (Figures 4 & 5). Overall, the average time from admission decision to VH was 61 minutes, and ACCU arrival was 115 minutes during the entire intervention period, with 41% (n = 33) of ACCU admissions arriving within the 60-minute target. ACCU constraints such as capacity, cleaning protocols, and staffing caused most delays (n = 69, 56%). Other delay reasons were VH coordination (n = 13, 19%), patient testing in the Clinic (n = 7, 10%), and multiple delay factors for some patients. Escalation of care events still decreased despite pandemic-related delays.

Regular meetings were held to receive interdisciplinary feedback on the new admission process. In consideration of prioritizing ACCU beds for Clinic acuity patients over other monitored beds, no systematic delays were noted for admissions from other Heart Center units. ACCU teams reported VH coordination as time-consuming, particularly in early implementation; however, Clinic and ACCU teams reported improved communication regarding admissions. Nursing reported increased knowledge of patient status and needs with full team participation in VH. We observed overall adherence to I-PASS in the Clinic. No handoff-related errors were reported during the implementation period.

## DISCUSSION

This work describes our Heart Center’s QI process of enhancing timeliness and reducing care escalation for cardiac patients pending admission to the ACCU from the Clinic. The benefits of specialized cardiovascular care for children with heart disease for pre-arrest prevention prioritize admission to cardiac specialty care rather than the emergency department.^13^ Growing capacity constraints, shorter inpatient stays, and sicker ambulatory patients necessitated this work. While there is a growing body of literature on the importance of structured handoffs for the movement of patients between inpatient, procedural, and emergency settings and to primary care post-discharge, there needs to be more information on handoffs from the ambulatory to the inpatient setting.^14,15^ The clinic is designed for high volume, short intervals of care, and rapid turnover of rooms rather than for extended monitoring or clinical interventions (ie, respiratory support).

The Institute for Health Improvement Model for Improvement provided a solid framework to define and describe the scope, develop metrics, implement I-PASS, and develop an admission process map.^8^ This systematic approach resulted in a standardized handoff process and improved patient safety, especially in hemodynamically fragile patients with higher rates of hospital readmissions than other pediatric populations.^16–18^ Use of I-PASS aligned with the hospital’s handoff policies and previously established team handoff model.

Interdisciplinary collaboration was essential across all Heart Center areas to understand the problem’s scope and identify solutions. Establishing a common language to identify, monitor, and prioritize admission for ill Clinic patients bolstered the success of this QI effort. Since its implementation, VH has been the platform for every patient admission discussion. Post-admission care escalation decreased with an increase in proactive ICU transfer planning for patients with decompensation risk. There were no post-admission CPR events after implementation. The average time to admission steadily decreased from the historical baseline but was negatively affected by COVID-19 pandemic restrictions and protocols. The onset of the COVID-19 pandemic disrupted almost all hospital processes and systems. Despite hindrances to admission efficiency during the pandemic, there was no impact on patient safety around admission. Timeliness for Clinic admissions to the ACCU achieved the goal of admission within 60 minutes from the decision to admit early in the experience. The ACCU capacity decreased drastically by 25% as double rooms were reduced to single rooms to support social distancing before the availability of vaccines and testing capabilities. Enhanced cleaning protocols increased the time for room turnaround between patients. The availability of COVID-19 screening of patients and their accompanying caregivers allowed return to pre-pandemic ACCU capacity, and time to admission further improved with the decrease in time for COVID-19 screening results.

Communication between Clinic and ACCU charge nurses enhanced transparency of admission timeliness, which in turn allowed both areas to plan for resource allocation, such as in-clinic monitoring and availability of ACCU admitting team readiness. Historically, Clinic patient handoff was provider-to-provider, leaving nursing team members in the Clinic and ACCU without sufficient patient details. Many Clinic patients complete ambulatory care without nursing involvement, limiting information in nursing handoff. Creating the VH is an example of handoff adaptation unique to the ambulatory setting, placing the sending and receiving teams on the same telephone/video line for I-PASS handoff. This intervention accommodated both Clinic and ACCU providers who exist within different areas of the hospital campus and have responsibilities away from the patient pending admission. The VH provided a safety net for Clinic nurses performing in-clinic monitoring and assessment, armed with patient information needed to understand the expected clinical trajectory and reason for concern. Both teams on the VH received the same information at one time with the opportunity to verbalize synthesis of received information, thereby increasing the reliability of patient handoff. Discussion of the appropriate level of care between both teams at the VH resulted in fewer transfers of care soon after admission, a resource-intensive risk from handoff failure, and less urgency to the transfers when they occurred.

This QI project demonstrated a sustained change in practice within the Clinic, enhancing patient safety. CHEWS became the standardized measure for clinical deterioration within the Clinic. High-risk patients, previously tracked by the busy Clinic team, now undergo structured monitoring with vital signs every 30 minutes. After an initial startup period, all acuity patients received in-clinic nurse monitoring, reliably documenting CHEWS scores congruent with scores upon admission. The establishment of communication processes empowered nurses to raise concerns if a patient’s CHEWS score increased and/or the patient’s condition changed. Discussing potential acuity patients at morning huddles increases situational awareness and planning throughout the Heart Center.

In the setting of ~50 cardiologists practicing within the Clinic, culture change and consistency are challenging.^19^ Established providers default to the more familiar, pre-intervention admission processes. Re-education, support from nursing and provider leaders, and visual references and cues in key charting locations facilitated the adoption of the new process.^19^ To achieve in-clinic monitoring, Clinic nursing staff increased daily. Room availability for vitals monitoring requires on-spot decision-making and reallocation of space for providers in the Clinic. The greatest challenge is keeping the daily clinicians involved in ACCU admissions attuned to acuity patient needs to ensure timely admission and reduced risk of decompensation in the Clinic.

This report is limited to one clinical department at a single institution; however, the process followed may provide other clinical areas and institutions a road map for developing their admission handoff processes from ambulatory to inpatient locations. Applicability and generalizability to other institutions and cardiac centers have yet to be discovered. Escalation of care events was rare in pre- and post-intervention cohorts; the sample was too small to achieve statistical significance. The quality of I-PASS handoffs specific for Clinic to ACCU was not assessed, but overall trends in I-PASS adherence were high in those clinical areas.

## CONCLUDING SUMMARY

This is the first article describing a standardized I-PASS handoff from an ambulatory to an inpatient setting. The Heart Center established sustainable, reliable handoff processes, in-clinic monitoring, and standardized admission processes for every patient admitted from the Clinic to the ACCU. There was a reduction in escalation of care events within 24 hours of ACCU admission. The development of critical language for ill patients in the Clinic allowed shared mental models and transparency across Heart Center teams for situational awareness and admission prioritization. Initial timeliness of admission improved, although COVID-19 negatively impacted it without causing an impact on patient safety. Team handoff using I-PASS is now the gold standard for handoff communication for Clinic admissions at this Heart Center.

## Supplementary Material


