# Supplementary material for: Enhanced Safety and Efficiency of Ambulatory Cardiology Admissions: A Quality Improvement Initiative
Source: Pediatr Qual Saf. 2024 May 9;9(3):e726. doi: 10.1097/pq9.0000000000000726 (PMC11093579; doi:10.1097/pq9.0000000000000726)
Supplement: Supplementary file 1 [file pqs-9-e726-s001.pdf]

Children's Hospital Early Warning Score (CHEWS) Reference Tool

Children's Hospital Boston

Children's Hospital Early Warning Score					
	0	1	2	3	Score
**Behavior/Neuro**	° Playing/sleeping appropriately<br>° Alert at patient's baseline	° Sleepy, somnolent when not disturbed	° Irritable, difficult to console<br>° Increase in patient's baseline seizure activity	° Lethargic, confused, floppy<br>° Reduced response to pain<br>° Prolonged or frequent seizures<br>° Pupils asymmetric or sluggish	
**Cardiovascular**	° Skin tone appropriate for patient<br>° Capillary refill ≤ 2 seconds	° Pale<br>° Capillary refill 3-4 seconds<br>° Mild* tachycardia<br>° Intermittent ectopy or irregular heart rhythm (not new)	° Grey<br>° Capillary refill 4-5 seconds<br>° Moderate* tachycardia	° Grey and mottled<br>° Capillary refill >5 seconds<br>° Severe* tachycardia<br>° New onset bradycardia<br>° New onset/increase in ectopy, irregular heart rhythm or heart block	
**Respiratory**	° Within normal parameters<br>° No retractions	° Mild* tachypnea/<br>° Mild increased WOB (flaring, retracting)<br>° Up to 40% supplemental oxygen via mask<br>° Up to 1L NC > patient's baseline need<br>° Mild* desaturation (< 5 below patient's baseline)<br>° Intermittent apnea self-resolving	° Moderate* tachypnea<br>° Moderate increased WOB (flaring, retracting, grunting, use of accessory muscles)<br>° 40-60 % oxygen via mask<br>° 1-2 L NC > patient's baseline need<br>° Nebs q 1-2 hr<br>° Moderate* desaturation (< 10 below patient's baseline)<br>° Apnea requiring repositioning or stimulation	° Severe* tachypnea<br>° RR below normal for age*<br>° Severe increased WOB (i.e. head bobbing, paradoxical breathing)<br>° >60 % oxygen via mask<br>° > 2 L NC > patient's baseline need<br>° Nebs q 30 minutes – 1 hr<br>° Severe* desaturation (<15 below patient's baseline)<br>° Apnea requiring interventions other than repositioning or stimulation	
**Staff Concern**		Concerned			
**Family Concern**		Concerned or absent			
				Total Score	

***Please refer to Vital Sign Reference Tool, the CHEWS Heart Rate and Respiratory Rate Reference Tool, and the Electronic Physiological Bedside Monitoring Policy**

		Mild	Moderate	Severe
**Respiratory Rate and Heart Rate**	**Infant**	≥ 10% ↑ for age	≥ 15% ↑ for age	≥ 25% ↑ for age
	**Toddler and Older**	≥ 10% ↑ for age	≥ 25% ↑ for age	≥ 50% ↑ for age
**Desaturation from patient's baseline O2 saturation**	**All ages**	5 points	10 points	15 points

© Children's Hospital Boston, 2024 All rights reserved • Publication Date 09/23/11
